# Comparative Binding Affinities of Flavonoid Phytochemicals with Bovine Serum Albumin 

**Published:** 2014

**Authors:** Shuqing Liu, Chunmei Guo, Yimeng Guo, Hongshan Yu, Frederick Greenaway, Ming-Zhong Sun

**Affiliations:** a *Department of Biochemistry, Dalian Medical University, Dalian,116044, China. *; b *Department of Biotechnology, Dalian Medical University, Dalian,116044, China. *; c *School of Bioengineering, Dalian Polytechnic University, Dalian 116034, China. *; d *Carlson School of Chemistry and Biochemistry, Clark University, Worcester, MA 01610, USA *

**Keywords:** Flavonoids, Bovine serum albumin, Binding capacity, Structure, Function

## Abstract

Dietary flavonoids show beneficial effects in the prevention of chronic diseases. However, flavonoid bioavailability is poor, probably due to their interaction with serum albumins. In the current work, the binding interactions of eight related flavonoids, sharing a similar core structure, with bovine serum albumin (BSA) were investigated by fluorescence spectroscopy. The binding affinities of the flavonoids with BSA were in the order hesperetin (K_A_=5.59 × 10^5^)> quercetin (4.94 × 10^5^) > naringenin (3.04 × 10^5^) > isoquercitrin (4.66 × 10^4^) > icariin (3.60 × 10^4^) > rutin (1.65 × 10^4^) > hesperidin (2.50 × 10^3^) > naringin (8.70 × 10^2^). The associations of specific structural components of the flavonoids with their binding properties to BSA were also explored and hydrophobicity, functional group substituents, steric hindrance effects and the spatial arrangements of substituents seem to be the key factors for the affinities of flavonoids towards BSA. The results from the current work contribute to a better understanding of the transport of flavonoids in plasma and helping predict their physiological functions based on their intrinsic structures.

## Introduction

Serum albumin (SA) is the most abundant protein in plasma. It is a single non-glycosylated protein of 585 amino acids with three homologous domains I, II and III. Each domain is composed of two separate subdomains A and B connected by random coils (1-3). SA acts as a depot and carrier of endogenous and exogenous compounds such as fatty acids, amino acids and pharmaceuticals (4-6). The binding capabilities of physiological molecules with SA are largely due to two major hydrophobic binding sites located within particular cavities in subdomains IIA and IIIA (7, 8). The drug-SA interaction plays a critical role in drug disposition and efficacy. The bound drug acts as a depot while the unbound drug produces the desired pharmacological effect. The drug-SA complexes dissociate to replenish the free drug that is removed by metabolic or elimination processes, which prolongs the duration of the drug action (9-11). The drug-SA binding affinity is the determining factor for the therapeutic, pharmacodynamic and toxicological properties of drugs (12-15). Bovine serum albumin (BSA) is one of the most extensively studied proteins due to its high structural homology with human serum albumin (HSA). The stable BSA-drug complex is an ideal model for gaining fundamental insights into HSA-drug and plasma-drug binding.

Flavonoids are important phytonutrient components widely distributed in plant foods. They have a wide range of biochemical and pharmacological properties, including antiplatelet, antioxidant, antiradical, anticarcinogenic, antiviral, antimicrobial, antithrombotic and antimutagenic activities (16, 17). Flavonoids are divided into flavonol, flavone, isoflavone, flavanone, flavanol and anthocyanidin classes depending on their molecular substitutes. The polyphenolic flavonoids share a 15-carbon skeleton core structure (represented as C_6_-C_3_-C_6_) consisting of two phenyl (chromanol) rings linked through a pyran ring (18-20). Considerable epidemiological evidence indicates that the intake of dietary favonoid phytochemicals reduces the incidence of and the mortality of degenerative diseases (21, 22). More attention has been attracted to flavonoids as they show great potential value in human health care. However, their bioavailability is often limited due to interaction with their transporter, SA (23, 24). The distribution, duration and intensity of physiological actions of flavonoids in the human body, as well as their metabolism and elimination, are correlated with their affinities towards SA (25, 26). Thus, investigation of the binding of flavonoids with SA is of fundamental importance and can help provide insights for new drug development.

Structural variations of flavonoids can affect their binding capacibilities to SA (27, 28). In this study, we investigated the binding affinities of quercetin, rutin, isoquercitrin, icariin, hesperetin, hesperidin, naringenin and naringin, which have similar structures, with BSA by fluorescence spectroscopy. The results indicated that minor modifications and/or arrangements of the functional groups of flavonoids affected their binding affinities with BSA. The results provide bases for estimating/evaluating the contributions of different functional groups of flavonoids to their binding with BSA. Results from current work give a better understanding of the importance of various structural components of flavonoids on their transport in plasma, and could help in the selection of effective dietary flavonoid drugs for the prevention and treatment of human diseases.

## Experimental

*Materials and instruments*

Quercetin, isoquercitrin, rutin, hesperetin, hesperidin, naringenin, naringin and icariin were generous gifts from Prof. Fengxie Jin of the School of Bioengineering, Dalian Polytechnic University, Dalian, China. Bovine serum albumin (BSA) with purity >98% was from Acros (Spain). All other chemicals were analytical grade from commercial sources. The HITACHI 650-60 fluorescence spectrometer was from Hitachi, Japan.

*Purity analyses of eight flavonoid phytochemicals by HPLC*

High performance liquid chromatography (HPLC) was performed to analyze the purity of flavonoid phytochemicals. Each of the eight flavonoids (0.55 mg) was dissolved separately in 2 mL of methanol and filtered through a 0.2 μm filter. For purity analysis, 10 μL of each flavonoid sample was analyzed by a Waters 2690/996 HPLC system using an Intersil ODS-3 C18 column (4.6 × 250 mm) at 25 °C. The mobile phase was 60% (v/v) methanol containing 0.2% phosphoric acid. HPLC elution was performed with a flow rate of 1.0 mL/min. The absorbance wavelength was 254 nm for quercetin, isoquercitrin, rutin, hesperetin and hesperidin, 283 nm for naringenin and naringin, and 270 nm for icariin. Triplicate measurements were performed for each sample. HPLC plots were collected and processed using Millennium 32 chromatographic software. 

*Binding assays of eight flavonoid phytochemicals with BSA*

A 10 μM BSA stock solution was prepared in 0.1 M Tris-HCl buffer (pH 7.4). Stock solutions (0.2 mM) were prepared of the eight flavonoids in dimethyl sulfoxide (DMSO) and were stored at 4 °C prior to use.

The binding capacities of flavonoids with BSA were investigated (2, 14). Briefly, a series of assay solutions was prepared by mixing 0.5 mL of 10 μM BSA with various volumes of the stock solution of each flavonoid, and diluting with 0.1 M Tris-HCl buffer (pH 7.4) to a total volume of 5 mL. The concentrations of quercetin, rutin, isoquercitrin, icariin, hesperetin, naringenin, hesperidin and naringin were in the ranges of 5.30×10^-7^-5.30×10^-6^ M, 8.30× 10^-7^-3.73×10^-6^ M, 1.55×10^-6^-5.18×10^-6^ M, 1.04×10^-6^-5.18×10^-6^ M, 1.04×10^-6^-5.18×10^-6^ M, 5.18× 10^-7^-5.18×10^-6^ M, 4.15×10^-7^-3.73×10^-6^ M and 8.30×10^-7^-4.15×10^-6^ M, respectively. In each tube, the concentration of BSA was constant while the concentration of flavonoids was different. The resultant mixtures were incubated at room temperature for 1 h and transferred into a quartz cuvette for analysis by fluorescence spectroscopy. Fluorescence spectroscopy was carried out at 25 ^o^C with a HITACHI 650-60 spectrometer (Hitachi, Japan) interfaced to a microcomputer. The excitation wavelength was 280 nm and the emission wavelength was 340 nm, and the excitation and emission slit widths were both set at 5 nm. 

The stereospecific interaction between flavonoid and BSA is quantified by the apparent association constant (K_A_) as previously reported (5, 14). For the binding of BSA with a flavonoid: 

Equation (1) $$\mathrm{nD}+P\underset{\Leftrightarrow }{K_{A}}D_{n}P$$

Where P is BSA, D is flavonoid, D_n_P is the complex and n is the number of flavonoid binding sites, and the equilibrium constant, K_A_ is given by:

Equation (2) $$K_{A}=\frac{\left[D_{n}P\right]}{{\left[D\right]}^{n}[P]}$$

Equation (3) $$\log K_{A}=\log\frac{\left[D_{n}P\right]}{{\left[D\right]}^{n}[P]}$$

As the D_n _P complex is:

Equation (4) $$\frac{\left[P\right]}{\left[P\right]+[D_{n}P]}=\frac{I}{I_{0}}$$

So the quenching equation is:

Equation (5) $$\log K_{A}=\log\frac{∆I}{I}-\mathrm{nlog}[D]$$

Where [D] is the concentration of flavonoid, I_0_ is the fluorescence intensity in the absence of flavonoid, I is the fluorescence intensity in the presence of flavonoid, and ΔI = I_0_ - I. K_A_ and n can be calculated from equation (5). 

*Data statistical analysis*

Triplicate assays were performed for each experiment. The data were expressed as the mean ± standard deviation (S.D.). SPSS 13.0 software was used for all statistical analyses. One-way analysis of variance (ANOVA) was used to determine the significant differences in two comparisons. Statistical significance was set at *P*<0.05.

## Results and Discussion

*Structures of flavonoid phytochemicals*

**Figure 1:**
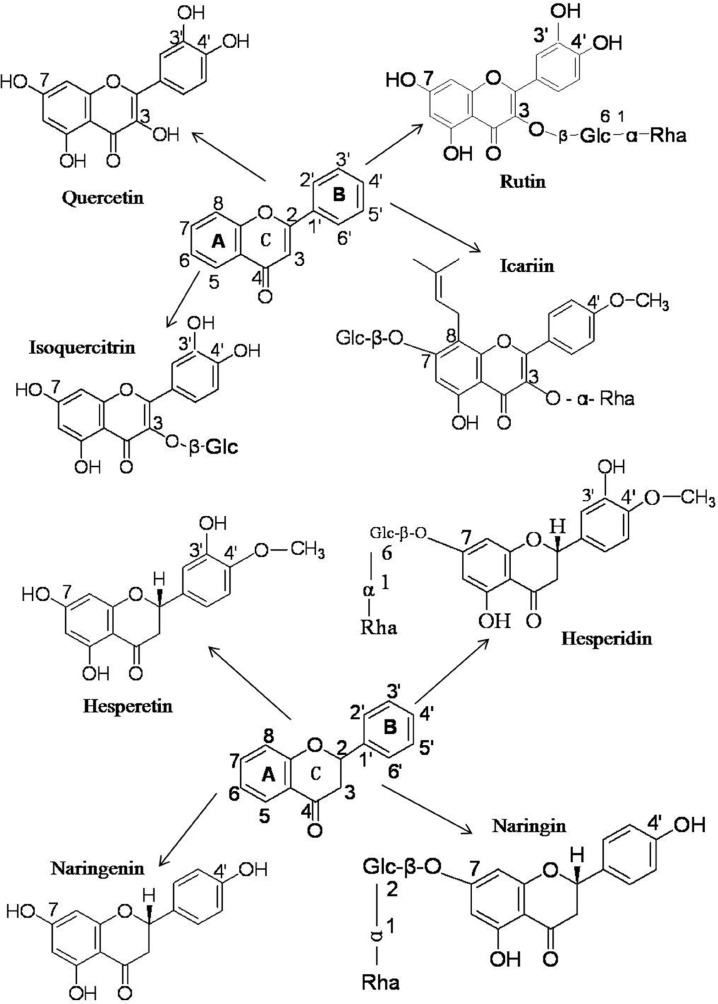
The chemical structures of quercetin, rutin, isoquercitrin, icariin, hesperetin, hesperidin, naringenin and naringin

The flavonoids utilized the current work can be cataloged as flavonols (quercetin), and flavones (rutin, isoquercitrin and icariin), which all share the core structure of flavone and flavanones (hesperetin, hesperidin, naringenin and naringin) (Figure 1). On one hand, these flavonoids share a similar structure, but on the other hand they also have their individual intrinsic structures (Figure 1). The structural differences among these flavonoids might result in and explain their functional differences. 

*Purity analyses of flavonoid phytochemicals*

**Figure 2:**
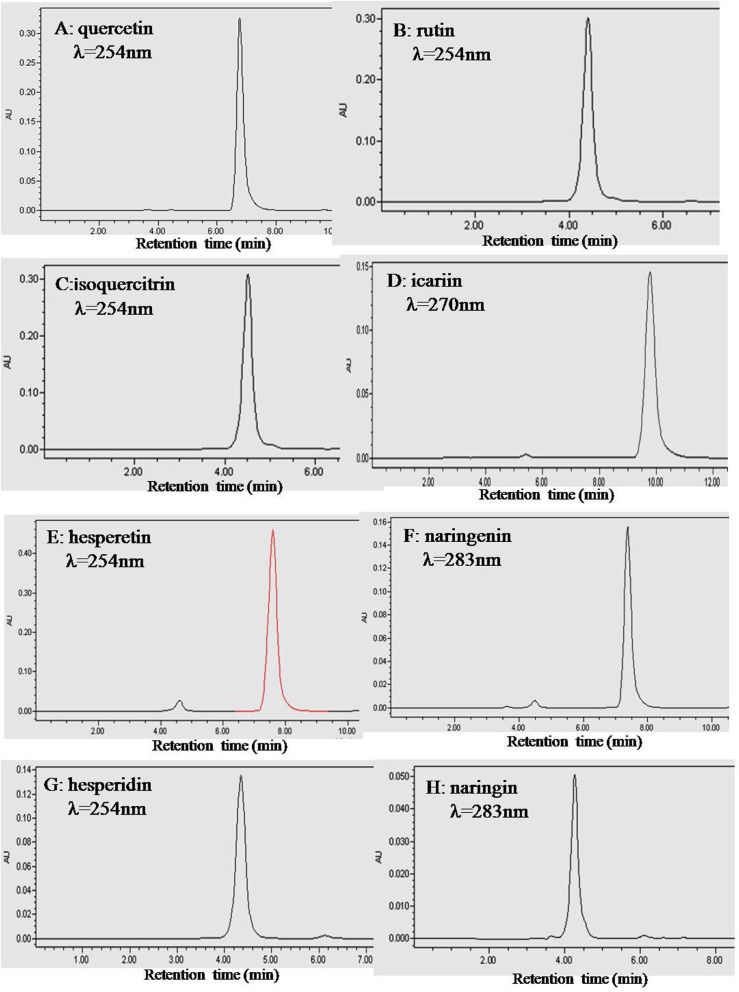
HPLC chromatograms of the eight flavonoids. Plots A, B, C, D, E, F, G and H are for quercetin, rutin, isoquercitrin, icariin, hesperetin, naringenin, hesperidin and naringin, respectively. HPLC was performed with an Intersil ODS-3 C18 column (4.6 × 250 mm) at 25 ^o^C. The mobile phase was 60% (v/v) methanol containing 0.2% phosphoric acid. The column was eluted with a flow rate of 1.0 mL/ min. The absorbance wavelength was set at 254 nm for quercetin, rutin, isoquercitrin, hesperetin and hesperidin, 283 nm for naringenin and naringin, and 270 nm for icariin.

To ensure that the fluorescence results we obtained were indeed due to the flavonoid, a purity analysis was carried out. The HPLC chromatograms of quercetin, rutin, isoquercitrin, icariin, hesperetin, naringenin, hesperidin and naringin are shown in Figure 2. The peak of each individual flavonoid dominated its chromatogram and the calculated purities of the eight flavonoids were over 95% based on both the peak area and intensity height (Table 1). 

**Table 1:** HPLC Determinations of the purities of the eight flavonoid phytochemicals

**Flavonoids**	**N** a	**Wavelength (nm)** b	**Retention time (min)**	**Purity (area) (%)** c	**Purity (height ) (%)** d
Quercetin	3	254	6.75 ± 0.01	98.22 ± 0.02	98.49 ± 0.02
Isoquercitrin	3	254	4.51 ± 0.01	97.12 ± 0.02	97.20 ± 0.02
Rutin	3	254	4.40 ± 0.01	99.33 ± 0.03	99.33 ± 0.02
Naringenin	3	254	7.38 ± 0.02	95.64 ± 0.02	95.10 ± 0.02
Naringin	3	254	4.26 ± 0.01	95.06 ± 0.04	95.31 ± 0.03
Hesperetin	3	283	7.83 ± 0.02	95.85 ± 0.03	95.02 ± 0.03
Hesperidin	3	283	4.35 ± 0.02	96.53 ± 0.02	96.78 ± 0.03
Icariin	3	270	9.78 ± 0.02	96.85 ± 0.03	96.29 ± 0.03

a N = the number of measurements per experiment;

b The wavelength used for monitoring the elution of flavonoid phytochemicals by HPLC;

c The purities of eight flavonoid phytochemicals were calculated based on their peak areas;

d The purities of eight flavonoid phytochemicals were calculated based on their peak heights.

*Binding of flavonoid phytochemicals to BSA*

**Figure 3:**
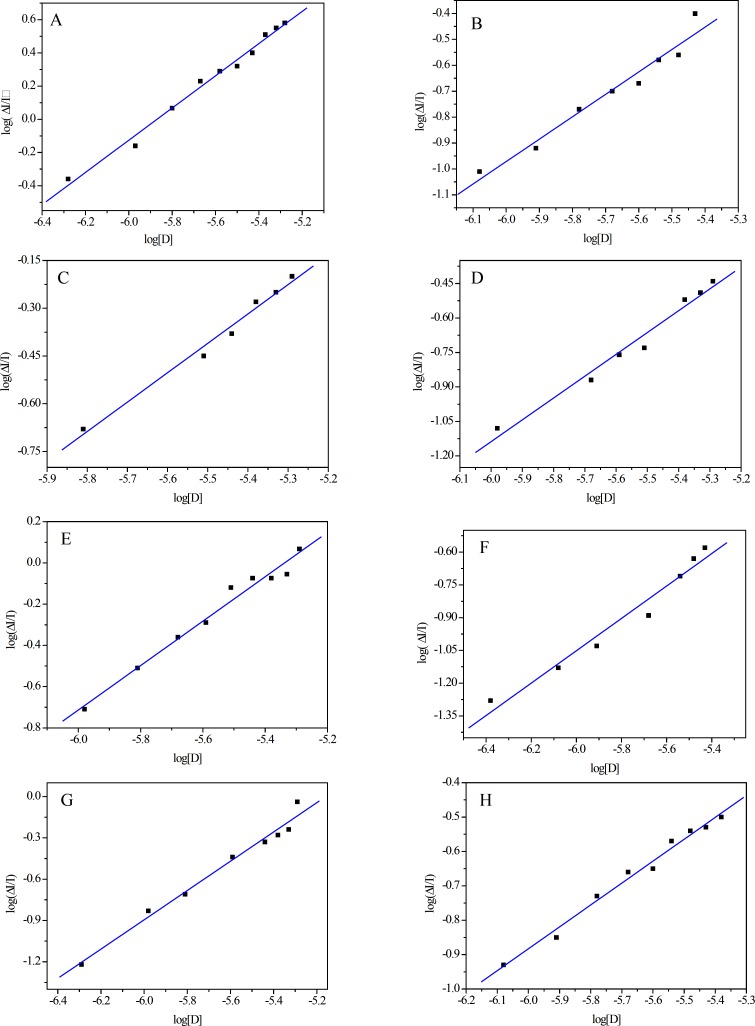
Double-logarithm curves of BSA fluorescence for flavonoids. A, B, C, D, E, F, G and H are the plots of log (ΔI/I) *versus *log [D] of quercetin, rutin, isoquercitrin, icariin, hesperetin, naringenin, hesperidin and naringin with the concentration ranges of 5.30×10^- 7^-5.30×10^-6^ M, 8.30×10^-7^-3.73×10^-6^ M, 1.55×10^-6^-5.18×10^-6^ M, 1.04×10^-6^-5.18×10^-6^ M, 1.04×10^-6^-5.18×10^-6^ M, 5.18×10^-7^-5.18×10^-6^ M, 4.15×10^-7^-3.73×10^-6^ M and 8.30×10^-7^-4.15×10^-6^ M, respectively. Significant differences exist for all comparisons between any two individual flavonoids (P<0.05) by SPSS 13.0 statistical analysis.

Epidemiological evidence indicates that a diet rich in flavonoids reduces the incidence of chronic diseases such as cardiovascular diseases, diabetes, cancers, neuronal diseases and stroke (29, 30). However, whether flavonoids play a significant role in preventing such chronic diseases has been questioned because of their low bioavailability (31). The interactions of flavonoids with SA determine their bioavailability and toxicology. BSA-flavonoid binding has been investigated by analyzing the perturbation of the intrinsic BSA fluorescence due to its Trp residues, which is observed by excitation at a wavelength of 280 nm (5, 14, 15). The fluorescence emission intensity of BSA has been observed to decrease as flavonoid concentration increases until at a 1:1 BSA:flavonoid ratio the complex is not fluorescent. Thus flavonoid molecules quench the Trp fluorescence, indicating that they bind near the Trp position (5, 14, 15). Our results indicate that as the flavonoid concentration increases, the fluorescence intensity of BSA decreases remarkably, implying that flavonoids we have studied also bind with BSA at or near the Trp residues. Figure 3 shows the plots of log ΔI/I *versus *log [D] for quercetin, rutin, isoquercitrin, icariin, hesperetin, naringenin, hesperidin and naringin for concentrations, [D], between 0.5 μM and 5 μM.

The K_A _values and the number (n) of binding sites for all eight flavonoids were calculated and are summarized in Table 2. Hesperetin had the greatest K_A_ for binding with BSA (5.59×10^5^), followed by quercetin (4.94×10^5^), naringenin (3.04×10^5^), isoquercitrin (4.66×10^4^), icariin (3.60×10^4^), rutin (1.65×10^4^), hesperidin (2.50×10^3^) and naringin (8.70×10^2^). SPPS 13.0 analysis indicated that the differences of binding activities of all eight flavonoid phytochemicals used in the current work are statistically significant (*P*<0.05). For all eight flavonoids the number of BSA binding sites was close to 1 (Table 2).

**Table 2:** The number of binding sites (n) and apparent association constants (K_A_) of eight flavonoid phytochemicals

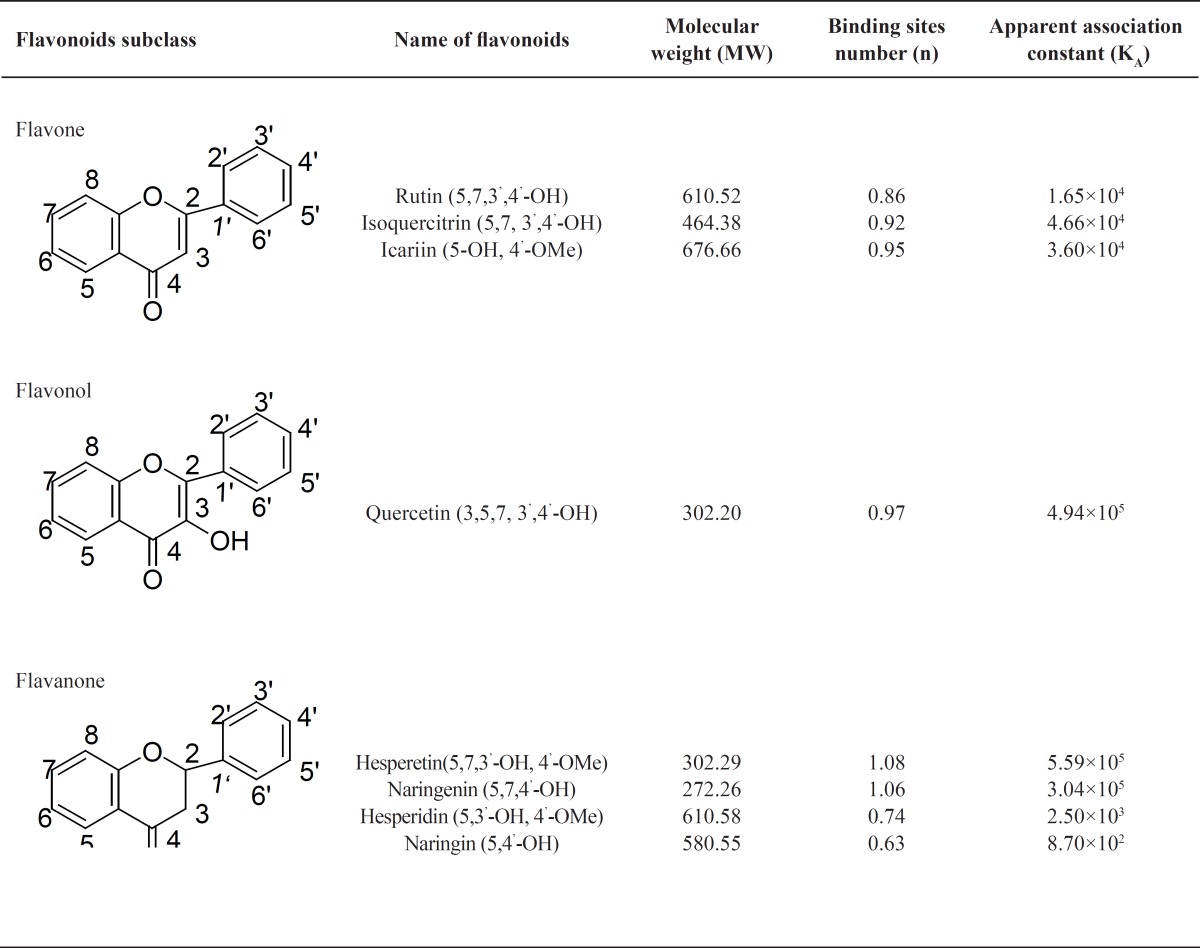

The binding capacities of flavonoids with BSA are determined by the specifics of their structures. Rutin and isoquercitrin share the same core flavone structure with quercetin but have no free 3-OH group on the C-ring due to their O-Glc-α-Rha and O-β-Glc substituents (Figure 1), which leads to a decrease in the BSA binding association constant, K_A_, from 4.94 × 10^5^ to 4.66 × 10^4^ and 1.65 × 10^4^, respectively. A similar phenomenon was also observed for hesperetin and hesperidin, and for naringenin and naringin. When the free 7-OH groups in the A-ring of hesperetin and naringenin were replaced by 7-O-β-Glc(6→1)-α-Rha and 7-O-β-Glc(2→1)-α-Rha to become hesperidin and naringin, respectively, their BSA binding association constants decreased from 5.59×10^5^ to 2.50×10^3^ and from 3.04×10^5^ to 8.70×10^2^. Considering that the only structural differences between hesperidin and hesperetin, and naringin and naringenin are the replacement of the 7-OH group by 7-O-β-Glc (6→1)-α-Rha and 7-O-β-Glc(2→1)-α-Rha, it is apparent that the presence of the free 7-OH enhances the binding capacities of flavanones with BSA. The hydroxyl group can interact with amino acid residues of BSA to form hydrogen bonds to improve the binding affinities. When the free -OH is replaced by saccharides, the molecular size increases and the resultant steric hindrances in the binding pocket result in a lower binding affinity to BSA. Furthermore, the additional polar saccharide groups render these molecules less hydrophobic, and could affect their orientation in relation to the hydrophobic environment of BSA (32-38). Thus substitution of the 3-OH and 7-OH groups has marked effects on the binding affinities of flavonoids with BSA.

When the 3-OH in the C-ring of quercetin was replaced by 3-O-Glc-α-Rha in rutin and 3-O-β-Glc in isoquercitrin, the binding affinities to BSA decreased by one order of magnitude, but when the 7-OH groups in the A-rings of hesperetin and naringenin were replaced by 7-O-β-Glc(6→1)-α-Rha and 7-O-β-Glc(2→1)-α-Rha in hesperidin and naringin, the binding affinities dropped by two orders of magnitude. 

The result indicates that substituents on the hydroxyl group of C_7_ in the A ring are more important for flavonoid interaction with BSA than are substituents on the hydroxyl group of C_3_ in the C-ring and thus that the A ring might be the more critical structure for binding to the hydrophobic region of BSA. 

In comparison, the binding affinity of icariin with BSA was much higher than of hesperidin and naringin. The difference in affinity can be ascribed to the 7-O-β-Glc in the A ring of icariin being smaller than the 7-O-Glc-α-Rha of hesperidin and naringin. In addition, the 8-alkyne of icariin enhances its lipid solubility and lipid-soluble drugs are more likely to enter the hydrophobic cavity of BSA and form a drug- BSA complex (33, 34, 36, 37). The two effects combine to contribute to the higher binding affinity of icariin with BSA. 

Taken together, the current work indicates that the binding capacities of flavonoid phytochemicals with BSA are related to their structures. The above results agree with published data for the binding affinities of other kinds of flavonoids with BSA, for which hydrogen bonding, hydrophobic forces and steric hindrance effects have been considered to be the driving forces for BSA-flavonoid association (3, 9, 14, 27, 28, 32-38). The study of the interactions of quercetin, rutin, hyperin and baicalin with HSA, indicated that flavonoids containing the groups C(3)–OH and C(4)=O, or C(5)–OH and C(4)=O, or C(3′)–OH and C(4′)– OH could form flavonoid-HSA complexes (9, 14, 28, 32). The numbers of hydroxyl groups on the A and B-rings of flavonoids affected the binding affinities and the number of binding sites of flavonoids with BSA significantly. Increasing numbers of hydroxyl groups on the A- and B-rings increased binding affinities of flavonols with BSA, presumably by forming hydrogen bonds. BSA has hydrophobic groups in the interior of its tertiary structure and polar groups at its surface. Hydrogen bonds may take place between –OH groups of flavonoids and polar groups at the BSA surface (35, 36). Glycosylation of their hydroxyl groups decreased the binding affinities of flavonoids with proteins (37, 38), as confirmed by our results. Glycosylation increases the polarity of the molecule and this lessens the ability of the flavonoid to penetrate into the Trp-rich hydrophobic interior regions of BSA. Glycosylation also increases the size of the molecule and steric hindrance effects in the binding pocket may also weaken the binding affinity of the flavonoid with BSA. Finally, glycosylation decreases the hydrophobicity of flavonoids, reducing their binding affinity with BSA (37, 38). 

## Conclusion

In spite of relatively small differences in the chemical structures of the eight flavonoids studied, large differences were observed in their binding affinities to BSA. The binding affinities of flavonoids are closely associated with their intrinsic structures in that minor modifications/arrangements of the functional groups affect the binding of flavonoids to BSA. Relative hydrophobicities, the presence of 3-OH and 7-OH groups, and steric hindrance effects seem to be the key factors determining the affinities of flavonoids towards BSA. The current work facilitates understanding of the structural bases of flavonoid transport in plasma and assists the prediction of their physiological behaviors.
